# Detection of Cathelicidin-1 and Cathelicidin-2 Biomolecules in the Milk of Goats and Their Use as Biomarkers for the Diagnosis of Mastitis

**DOI:** 10.3390/ani15152301

**Published:** 2025-08-06

**Authors:** Maria V. Bourganou, Dimitra V. Liagka, Konstantinos Vougas, Daphne T. Lianou, Natalia G. C. Vasileiou, Konstantina S. Dimoveli, Antonis P. Politis, Nikos G. Kordalis, Efthymia Petinaki, Vasia S. Mavrogianni, George Th. Tsangaris, George C. Fthenakis, Angeliki I. Katsafadou

**Affiliations:** 1 Faculty of Public and One Health, University of Thessaly, 43100 Karditsa, Greece; mbourganou@uth.gr; 2 Faculty of Animal Science, University of Thessaly, 41110 Larissa, Greece; dliagka@uth.gr (D.V.L.);; 3 Proteomics Research Unit, Biomedical Research Foundation of the Academy of Athens, 11527 Athens, Greece; kvougas@bioacademy.gr (K.V.); gthtsangaris@bioacademy.gr (G.T.T.); 4 Veterinary Faculty, University of Thessaly, 43100 Karditsa, Greece; dlianou@vet.uth.gr (D.T.L.); kdimoveli@uth.gr (K.S.D.); vmavrog@vet.uth.gr (V.S.M.);; 5 Private Veterinary Practice, Dimokratias Road, Ovria, 26500 Patras, Greece; apolitis.vet@gmail.com; 6 Private Veterinary Practice, Patron Road, 20130 Korinthos, Greece; nikolaoskordalis@gmail.com; 7 University Hospital of Larissa, 41110 Larissa, Greece; petinaki@uth.gr

**Keywords:** biomarker, cathelicidin-1, cathelicidin-2, diagnosis, goat, mastitis, proteomics, sheep, somatic cell counts

## Abstract

The objectives of the present work were as follows: (i) the detection of cathelicidin biomolecules in the milk of individual goats during the early stages of mastitis and their potential use for the diagnosis of mastitis at its early stage and (ii) the evaluation of the presence of cathelicidin proteins in the bulk-tank milk from goat and sheep farms. In an experimental study, after inoculation of *Staphylococcus simulans* into a mammary gland of goats, bacteriological and cytological examinations of milk samples, as well as proteomics examinations [two-dimensional gel electrophoresis analysis (2-DE) and matrix-assisted laser desorption/ionization time-of-flight mass spectrometer (MALDI-TOF MS) analysis] were performed sequentially, from 4 to 48 h post-challenge. Cathelicidin-1 and cathelicidin-2 were detected consistently in milk samples obtained throughout the study, and spot optical densities obtained from PDQuest v.8.0 were recorded. Associations were calculated between the presence of mastitis in a mammary gland at a given timepoint and the detection of cathelicidin proteins in the respective milk sample. All inoculated mammary glands developed mastitis, confirmed by the consistent bacterial isolation from milk samples and the increased somatic cell content therein. Spot optical density of cathelicidin proteins was higher than in samples from contralateral mammary glands. There was a significant association between the presence of mastitis in a mammary gland and the detection of cathelicidin biomolecules in the respective milk sample; the overall accuracy was 81.8% (95% confidence interval: 70.4–90.2%). In a field investigation, the presence of cathelicidin proteins was evaluated in the bulk-tank milk of 32 dairy goat and 57 dairy sheep farms. In this part of the work, no cathelicidin proteins were detected in any bulk-tank milk sample of goat, 0.0% (95% confidence interval: 0.0–10.7%), or sheep, 0.0% (95% confidence interval: 0.0–6.3%), farms.

## Simple Summary

Τhis study aimed to detect cathelicidin proteins in goat milk during the early stage of mastitis and to assess their potential use in diagnosis of the infection. Goats were inoculated with *Staphylococcus simulans* and milk samples were analyzed regularly for 48 h post-challenge. Cathelicidin proteins were consistently detected in milk from infected mammary glands; this detection was associated with presence of mastitis, with an overall diagnostic accuracy of 82%. The detection of cathelicidin proteins in the milk of individual animals occurred earlier than the increase in somatic cell counts in milk, an established method for detection of mastitis, indicating the advantages of the method for the early detection of the infection.

## 1. Introduction

An early report regarding the production of antimicrobial proteins by the epithelial cells in the mammary gland was published by McDonald et al. [1]; these researchers identified the isoform of serum amyloid in the colostrum of ewes and termed it ‘mammary-associated serum amyloid’. Later, Addis et al. [2,3] reported the production of cathelicidin proteins by the mammary epithelial cells during the early stage of intramammary infection. More recently, Katsafadou et al. [4] also suggested that mammary epithelial cells produced proteins (specifically haptoglobin and serum amyloid) implicated in the inflammation process during intramammary bacterial infection.

Cathelicidin biomolecules are synthesized in the epithelial cells of the mammary gland; they are released when these cells are exposed to invading pathogens [5]. Further, it has been indicated that the release of cathelicidin proteins occurred before the influx of leucocytes into the mammary gland [5]. Thus, they participate in the early stages of mammary defense against the invading pathogens [4]. Their presence in the early stages of intramammary infections makes these biomolecules valuable biomarkers for the early diagnosis of mastitis.

The benefits of early diagnosis of mastitis refer to the early instigation of appropriate treatment. That can lead to effective bacterial killing, before development of substantial lesions to the mammary tissue [6]. The hypothesis of Cubeddu et al. [5] was confirmed by Katsafadou et al. [7]; these authors reported the detection of cathelicidin-1 for the early detection of mastitis in ewes by employing standard diagnostic procedures. They also suggested that cathelicidin-1 could be detected in the milk of infected mammary glands of ewes before the increase in somatic cell counts in the respective glands [7].

In goats, studies have revealed the presence of cathelicidin-1, cathelicidin-2, cathelicidin-3, cathelicidin-6 and cathelicidin-7 in milk during mammary inflammation [8,9,10,11,12]. In general, those studies reported the presence of the proteins to be allied to the increase in somatic cell counts in milk, but did not focus on the potential detection of mastitis at its early stage. These studies were limited to investigations in individual animals, with no assessment of the detection of the proteins in the bulk-tank milk produced at farm level. Notably, this facet was also not studied by researchers who had evaluated the presence of cathelicidin proteins in the milk of ewes

The objectives of the present work were as follows: (i) the detection of cathelicidin biomolecules in the milk of individual goats during the early stages of mastitis and their potential use for the diagnosis of mastitis at its early stage and (ii) the evaluation of the presence of cathelicidin proteins in the bulk-tank milk from goat and sheep farms.

## 2. Materials and Methods

### 2.1. Detection of Cathelicidin Proteins in the Milk of Goats After Intramammary Inoculation of Staphylococcus simulans

#### 2.1.1. Outline of the Experimental Study

**Figure 1:**
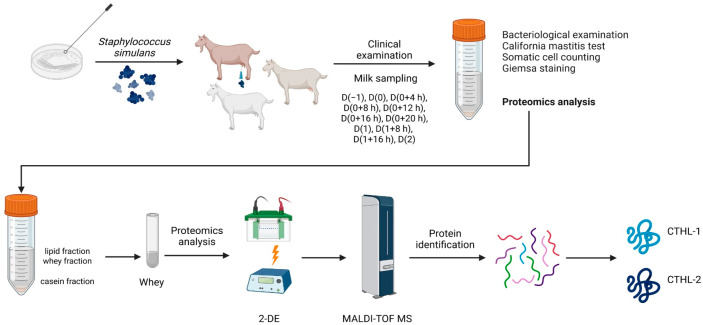
Summary of the work process in the experimental study (figure produced in BioRender, under academic publication licence no. EN28LJSRU7 (2025)).

Goats (*n* = 3) were inoculated with *S. simulans* directly into the gland cistern of the mammary gland. The organism is a confirmed mammary pathogen [13]. The small number of goats in this experiment was requested by the local animal welfare regulatory authorities, given that a similar study had been conducted previously in ewes. A summary of the process in the experimental study is depicted in Figure 1.

The intramammary inoculations were performed on the 5th day after parturition. Development of mastitis was confirmed by conventional diagnostic approaches. Thereafter, samples of mammary secretion were collected for proteomics analysis. The principal investigator (author M.V.B.), who carried out this analysis, was blinded to the origin of milk samples (i.e., from inoculated or control mammary gland or from a particular sampling point during the experiment).

The entire inoculation procedure was performed as described previously, using an established and standardized protocol [13]. The inoculum contained approximately 10^5^ colony-forming units of *S. simulans*, as determined by the method of Miles and Misra [14]. The bacterial suspension was injected directly into the gland cistern of the left mammary gland.

#### 2.1.2. Animal Examinations and Samplings

A detailed clinical examination of the goats, with special attention to the mammary glands, was performed twice before the challenge (on D(−1), i.e., the day before inoculation, and on D(0), i.e., the day of inoculation). Thereafter, clinical examinations were performed every four hours during the initial 24 h post-challenge (D(0+4 h), D(0+8 h), D(0+12 h), D(0+16 h), D(0+20 h), D(1)) and every eight hours during the subsequent 24 h post-challenge (D(1+8 h), D(1+16 h), D(2)). Hence, in total, nine samplings were performed post-inoculation.

At each of these time-points, a standardized clinical examination of the udder (observation, palpation, comparison between glands) was initially performed, always by the same clinician (author N.G.C.V.) [15].

Mammary secretion samples were collected aseptically and separately from each mammary gland (inoculated and uninoculated) of the goats, as detailed before [15].

#### 2.1.3. Conventional Laboratory Examinations

Samples were processed for bacteriological examination within 15 min. after collection, performed by conventional microbiological techniques. Bacteria were identified using established techniques [16,17]. In total, 66 samples were tested bacteriologically (11 samples from each mammary gland from each of three goats).

Moreover, all milk samples were also tested by means of the California Mastitis Test (CMT). The test was always carried out and scored by the same person (author D.V.L.). Five degrees of reaction (‘negative’, ‘trace’, ‘l’, ‘2’, ‘3’) were described [18].

For somatic cell counting, two sub-samples were produced from each mammary secretion sample and tested separately. Cell counting was performed by means of an automated counter (Lactoscan SCC; Milkotronic Ltd., Nova Zagora, Bulgaria). Milk smears were also prepared and air-dried. Leucocyte subpopulations were identified by direct microscopy after the staining of milk smears with Giemsa stain (Sigma-Aldrich, Burlington, MA, USA). In each case, 100 cells were observed and counted. In total, 132 samples were evaluated cytologically (66 samples as detailed above, from each of which two subsamples were obtained).

### 2.2. Evaluation of the Presence of Cathelicidin Proteins in the Bulk-Tank Milk from Goat and Sheep Farms

#### 2.2.1. Collection of Samples

In total, 32 dairy goat farms and 57 dairy sheep farms were included in this field investigation. Farms were included on a convenience selection, based on the willingness of farmers to accept a visit by the personnel of the University for collection of samples.

During the visit, the characteristics of the farms were recorded (management system applied on farms [19], number and breed of animals, month after kidding/lambing of does/ewes on farms, milking mode applied on farms). Samples were obtained from the milk bulk-tank of each farm, by using the aseptic technique, as described in detail before [20]; in total, four 20 mL milk samples were collected from the bulk-tank of each farm.

#### 2.2.2. Conventional Laboratory Examinations

Somatic cell counting and milk composition measurement were performed on the samples within 4 h after collection. Two of the samples were used for somatic cell counting and milk composition measurement and the remaining two were used for bacteriological examination. Two sub-samples were created and processed from each of the four samples, so that each separate test was performed four times. Initially, somatic cell counting (Lactoscan SCC; Milkotronic Ltd., Nova Zagora, Bulgaria) and milk composition measurement (Lactoscan Farm Eco; Milkotronic Ltd.) were performed.

Isolation of bacteria from the bulk-tank milk samples and their initial identification were performed using standard methods [16,17]. Detection of at least three confirmed staphylococcal colonies on at least one of the four agar plates cultured with each bulk-tank milk sub-sample from each farm was considered to confirm presence of the organism. The staphylococcal isolates were identified by using matrix-assisted laser desorption/ionization time-of-flight mass spectrometry (VITEK MS; BioMerieux, Marcy-l’-Étoile, France) [20].

### 2.3. Proteomics Analysis

All the milk samples were prepared for proteomics evaluation, as described in detail by Katsafadou et al. [4,7]. They were assayed by using two-dimensional gel electrophoresis analysis (2-DE), in accordance with the methodology described in detail by Anagnostopoulos et al. [21] and Katsafadou et al. [4,7].

Gel image analysis was performed as detailed by Katsafadou et al. [4,7]; spots with potential correspondence to cathelicidin proteins were studied in detail. The potential presence of the proteins was evaluated by assessing all spots in the region where these develop on the gels.

In the experimental work, the presence of cathelicidin proteins in milk samples collected from individual goats before or after challenge was evaluated. Protein identification was performed by peptide mass fingerprinting. Peptide mixtures were analyzed in a MALDI-TOF MS (matrix-assisted laser desorption/ionization time-of-flight mass spectrometer) (Ultraflex, Bruker Daltonics; Billerica, MA, USA). Matching of peptides and protein searches was carried out in the MASCOT Server 2 (Matrix Science; Boston, MA, USA). The method for protein identification was the same as the one followed by Katsafadou et al. [4,7].

Spot optical densities obtained from PD Quest v.8.0 for each spot of interest on each gel from the samples before or after challenge were recorded. In case of multiple spots indicative of the same protein, sums of optical densities of all spots were taken into account. The spot volume was used as the parameter for quantifying the protein expression.

### 2.4. Data Management and Analysis

All data were systematically recorded and organized using Microsoft Excel (versions 2405-2504) (Microsoft Corp.; Redmond, WA, USA).

Clinical mastitis was defined as the presence of clinically evident abnormal signs in the mammary glands of the goats. Subclinical mastitis was defined as the concurrent bacterial recovery from milk samples and the increased (>1.500 × 10^6^ cells mL^−1^) somatic cell counts therein; a secondary definition for subclinical mastitis was also given as the concurrent bacterial recovery from milk samples and the increased (≥‘2’) CMT score therein.

For the analysis, somatic cell counts were transformed to somatic cell scores (SCS), as described by Wiggans and Shook [22] and Franzoi et al. [23]. Following the assignment of numerical values to CMT scores and transformation of somatic cell counts to SCS, correlation between CMT scores and SCS was *r* = 0.928 (95% confidence interval (CI): 0.885–0.956) (*p* < 0.0001) and the corrected *r*^2^ was 86.1%.

Comparisons were made between challenged and control sides of the udder in the frequencies of (i) development of mastitis, (ii) isolations of *S. simulans* from samples, (iii) samples with increased somatic cell counts, and (iv) detection of cathelicidin proteins in the milk. Comparisons were also made between the detection of cathelicidin proteins and increased (>1.500 × 10^6^ cells mL^−1^) somatic cell counts in the milk of goats after challenge. The significance of associations was assessed by using Pearson’s chi-square test or the Fisher-exact test, as appropriate, and by taking into account all the sampling points of the experiment.

The Wilcoxon Signed Rank test was performed to evaluate differences in somatic cell counts and in spot optical densities of cathelicidin proteins in samples from inoculated or uninoculated sides of the udder. For the latter, separate evaluation was performed for each of the cathelicidin proteins detected.

The Spearman rank test was used to evaluate correlations between the spot densities of cathelicidin proteins detected; separate analysis was carried out for the challenged and the control glands. The Mann–Whitney test was employed to compare the median spot densities between the cathelicidin proteins detected throughout the study; it was also used to evaluate differences in the time post-challenge that cathelicidin protein or increased cell content were first seen in the milk samples. Through the use of the Kruskal–Wallis test, differences in spot optical densities were assessed between sampling points, separately for each of the proteins detected.

Accuracy measures (sensitivity, specificity, positive and negative predictive values, overall accuracy) were calculated to study the diagnosis of mastitis by means of the detection of cathelicidin proteins.

In the field investigation, with regard to the characteristics of the farms, basic descriptive analyses were performed. Exact binomial confidence intervals (CIs) were obtained. For continuous characteristics of farms, the normality of distribution was assessed by using the Shapiro–Wilk Test; separate analyses were carried out for goat and sheep farms.

Significance level was set at *p* < 0.05.

## 3. Results

### 3.1. Individual Animal Study

#### 3.1.1. Conventional Examinations

Before the challenge, both mammary glands of the goats included in the study appeared clinically healthy. No bacteria were isolated from any milk sample. Somatic cell counts were <0.500 × 10^6^ cells mL^−1^. In the Giemsa-stained milk films, only some macrophages (on average, three cells per 10 fields) were observed.

After inoculation, the experimental animals developed transient (for up to 16 h post-challenge) clinical signs in the inoculated side of the udder. Starting 4 h post-inoculation and until day D(2), *S. simulans* was consistently isolated in pure culture from samples from the inoculated mammary glands (in total, 27 recoveries from 27 samples). Cell content therein was found to progressively increase after challenge; cell counts in the inoculated glands were over 100% higher than in the control glands on D(0+16 h) and increased over 1.500 × 10^6^ cells mL^−1^ on D(0+20 h); further, CMT scores increased to ≥‘2’ on D(0+16 h) and thereafter. Neutrophils were predominantly seen in the milk films.

No uninoculated mammary gland developed clinical or subclinical mastitis (*p* < 0.0001 versus the inoculated glands). No bacteria were recovered from any milk sample from these glands (*p* < 0.0001 versus the inoculated glands). Neither cell content over 0.650 × 10^6^ cells mL^−1^ nor a CMT score ≥ ‘2’ was recorded in any sample (*p* < 0.0001 versus the inoculated glands).

#### 3.1.2. Proteomics Examinations

Before inoculation, no cathelicidins were detected in any milk sample from any goat.

After challenge, starting on D(0+4 h), in all goats, two cathelicidin biomolecules, specifically cathelicidin-1 and cathelicidin-2 (Figure S1), were consistently detected in samples from the inoculated mammary glands (for both proteins, in 27 of 27 samples; 100%). The proteins were also detected in milk samples from the uninoculated glands of two goats (cathelicidin-1) or of one goat (cathelicidin-2), starting on D(0+16 h) and continuing thereafter (in total, in 10 (cathelicidin-1) and 4 (cathelicidin-2) of 27 samples; 37.0% and 14.8%, respectively) (*p* < 0.0001 versus the inoculated glands).

**Figure 2:**
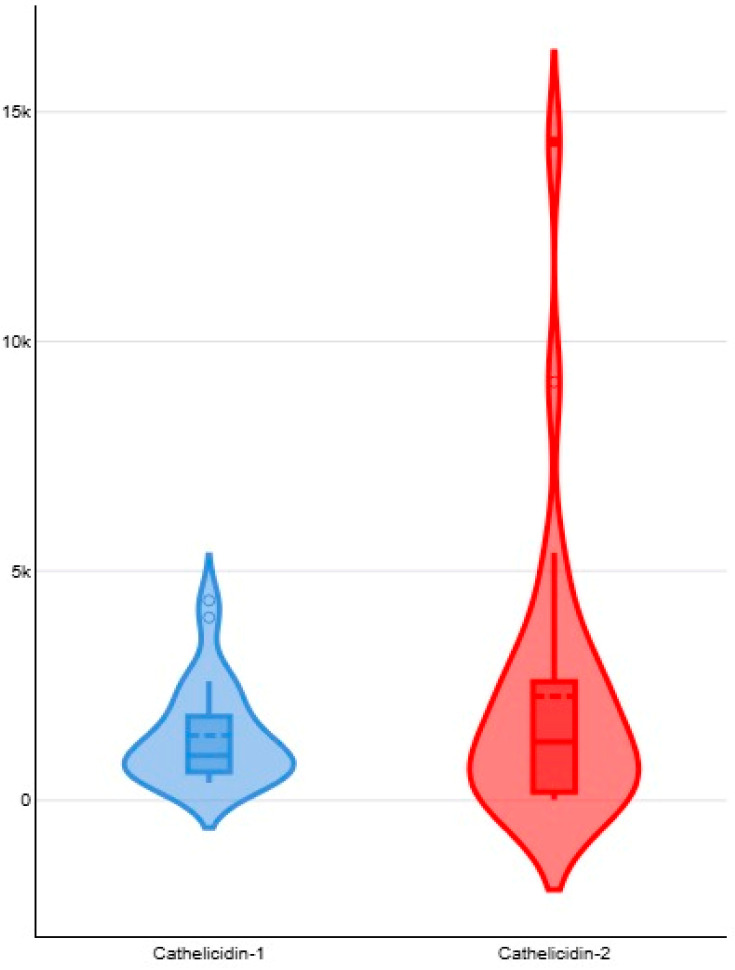
Violin plots for spot optical densities of cathelicidin-1 (blue plot) or cathelicidin-2 (red plot) on 2-DE gels obtained from sequential milk whey samples from mammary glands inoculated with *S. simulans* for 48 h subsequently to challenge; violin plots depict the probability density of the data at different values and compare probability distributions of the optical densities of the two cathelicidin proteins.

Throughout the study, differences in median spot optical densities of cathelicidin-1 or cathelicidin-2 in samples from inoculated versus uninoculated sides were significant: 976.6 (interquartile range (IQR): 1196.3) versus 0.0 (IQR: 24.2) for cathelicidin-1 and 1276.3 (IQR: 2355.4) versus 0.0 (IQR: 0.0) for cathelicidin-2 (*p* < 0.0001 for both comparisons) (Table 1). The difference in the median spot densities between cathelicidin-1 and cathelicidin-2 was not significant (*p* = 1.0) (Figure 2). Moreover, there was a clear correlation between the spot optical densities of cathelicidin-1 and cathelicidin-2 throughout the study for both the challenged (*r_sp_* = 0.688, *p* = 0.0008) and the control (*r_sp_* = 0.606, *p* = 0.0008) glands.

Median spot densities of cathelicidin-1 or cathlicidin-2 in samples from inoculated sides were significantly higher compared to before challenge (*p* = 0.0002 for both comparisons). Differences between sampling points were significant for cathelicidin-1 (*p* = 0.002), but not for cathelicidin-2 (*p* = 0.27).

Median spot optical densities in samples from uninoculated glands were not significantly different compared to before challenge (*p* > 0.30). Also, there were no significant differences between the sampling points after inoculation (*p* > 0.29).

#### 3.1.3. Detection of Cathelicidin Biomolecules for the Diagnosis of Mastitis

**Figure 3:**
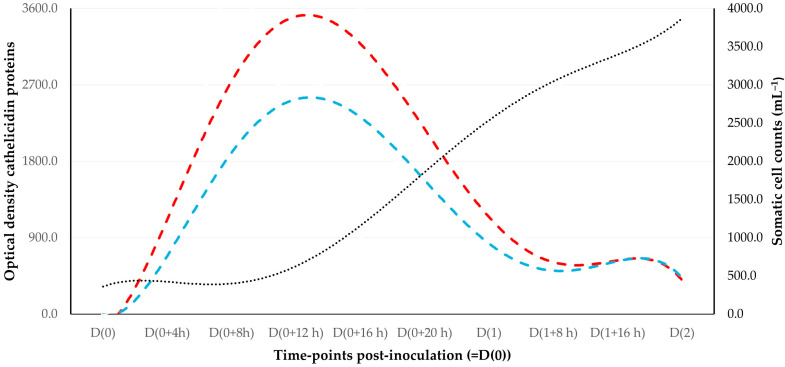
Trendlines for spot optical densities of cathelicidin-1 (blue dashed line) or cathelicidin-2 (red dashed line) on 2-DE gels obtained from sequential milk whey samples from mammary glands inoculated with *S. simulans* and comparison with trendline of somatic cell counts (black dotted line) in the same samples; trendlines for the spot optical densities of the two cathelicidin proteins indicated an early increase in the values, which reached maximum figures after 12 to 24 h post-challenge, in contrast with somatic cell counts, which increased at a later stage and continued to rise as the infection developed further.

Post-challenge, cathelicidins were identified in mammary secretion samples earlier than records of increased somatic cell counts (Figure 3) and increased CMT scores (Figure S2).

**Figure 4:**
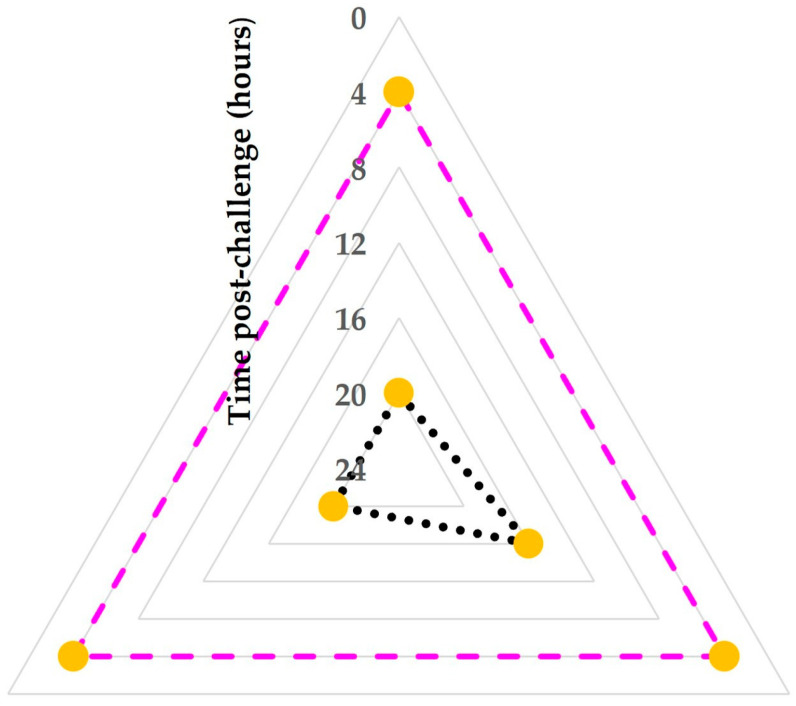
Radar-type plot of the time post-challenge, when cathelicidins (coloured dashed line) were first seen on 2-DE gels obtained from sequential milk whey samples from each of the three mammary glands inoculated with *S. simulans* and comparison with the time post-challenge when increased somatic cell counts (>1.500 × 10^6^ cells mL^−1^) or increased CMT scores (≥‘2’) (black dotted line) were first recorded in milk samples from the same glands (orange markers indicate the three mammary glands challenged in the study); the plot clearly indicates the early rise in cathelicidin proteins in the milk of inoculated animals (4 h post-challenge), in contrast to the substantially later development of the leucocyte response of the animals (≥16 h post-challenge) (grey lines are gridlines).

The median time of the first detection of cathelicidins in the milk of challenged mammary glands was 4 (interquartile range (IQR): 0) hours post-challenge. The median time that increased somatic cell counts (>1.500 × 10^6^ cells mL^−1^) and the median time that increased CMT scores (≥‘2’) in that milk were seen were 20 (IQR: 0) hours and 20 (IQR: 2) hours, respectively (*p* = 0.047 and 0.06, respectively, compared to the median time of the first detection of cathelicidins) (Figure 4).

Moreover, the difference between these frequencies from D(0+4 h) to D(0+16 h) was significant: 12/12 samples for detection of cathelicidin proteins versus 0/12 samples presence of increased somatic cell counts and 1/12 samples for increased CMT scores (*p* < 0.0001 for both comparisons) and 4/12 samples for somatic cell counts > 100% higher than in the contralateral gland (*p* = 0.0005). However, on D(0+20 h) and thereafter, there were no differences between the above frequencies (*p* = 1.0 for both comparisons).

There was a significant association between the presence of mastitis (clinical or subclinical) in a mammary gland on a sampling point and the detection of cathelicidin proteins in the respective milk sample (*p* < 0.0001) (Table 2). The sensitivity and the specificity of using the presence of cathelicidin proteins for the diagnosis of mastitis were 100% (95% CI: 86.3–100%) and 70.7% (95% CI: 54.5–83.9%), respectively. Positive and negative predictive values were 67.6% (95% CI: 56.4–77.0%) and 100% (95% CI: 88.1–100%), respectively. The overall accuracy of the detection of cathelicidin biomolecules for the diagnosis of mastitis was 81.8% (95% CI: 70.4–90.2%). There was clear evidence of correlation between results of the cathelicidin proteins’ detection and the presence of mastitis (*r_sp_* = 0.714, *p* < 0.0001); this correlation was stronger during the period of D(0+4 h) to D(1) (*r_sp_* = 0.714, *p* < 0.0001) than during the period D(1+8 h) to D(2) (*r_sp_* = 0.447, *p* = 0.06).

### 3.2. Farm Study

#### 3.2.1. Characteristics of Farms

The 32 goat farms were most frequently managed under the semi-extensive (*n* = 15, 46.9%) or semi-intensive (*n* = 10, 31.3%) system. The mean number of animals on farms was 296 ± 20 goats, mostly indigenous (*n* = 13, 40.6%) or crossbreed or Damascus (*n* = 5 each, 15.6%) breeds. The mean stage of lactation period of animals on the farms was 4.3 ± 0.4 months after kidding.

The 57 sheep farms were managed most frequently under the semi-intensive (*n* = 25, 43.9%) or semi-extensive (*n* = 20, 35.1%) system. The mean number of animals on the farms was 331 ± 19 sheep, mostly Lacaune (*n* = 19, 33.3%) or local (*n* = 15, 26.3%) breeds. The mean stage of lactation period of animals on the farms was 4.7 ± 0.3 months after lambing.

For both goat and sheep farms, the number of animals on the farms and the stage of lactation period of does or ewes on the farms followed normal distribution patterns (*p* > 0.12 for sheep and *p* > 0.20 for goat farms).

#### 3.2.2. Conventional and Proteomics Examinations

The details of the somatic cell counts and chemical composition in the bulk-tank milk of these farms are shown in Table 3. For both sheep and goat farms, somatic cell scores, fat content, and protein content followed normal distribution patterns (*p* > 0.07, >0.35 and >0.29, respectively). Staphylococci were isolated from the bulk-tank milk of 46.9% (95% CI: 30.9–63.6%) and 35.1% (95% CI: 24.0–48.1%) of goat and sheep farms, respectively.

No cathelicidin proteins were detected in any bulk-tank milk sample of goat: 0.0% (95% CI: 0.0–10.7%), or sheep: 0.0% (95% CI: 0.0–6.3%), farms.

## 4. Discussion

### 4.1. Individual Animal Study

The present results provide clear evidence that, in goats, the detection of cathelicidin biomolecules in milk is a useful and reliable biomarker that can be employed for the diagnosis of mastitis at its early stages. This study was designed as a targeted, high-throughput proteomics investigation of early molecular events in ovine mastitis by using a well-established challenge model. Therefore, it focuses on milk whey changes that accompany localized subclinical inflammation. The proteomics dataset is supported by confirmatory standard bacteriological findings and increased somatic cell counts, which together constitute the accepted diagnostic ‘gold standard’ for subclinical mastitis [24].

The advantage of the present results compared to findings of relevant studies performed previously is the collection of samples and the detection of the cathelicidin proteins within 4 to 8 h after infection. Indeed, no study has previously reported the kinetics of cathelicidin proteins in goat milk during the early stages post-infection. The presence of cathelicidin proteins in milk before the increase in somatic cell counts in milk (i.e., before the intramammary influx of leucocytes) is indicative of the rapidness of the response of the animals to the intramammary invasion of pathogens.

There is a notable difference to a similar study that we performed in sheep some time ago. In that animal species, cathelicidin-1 was found to be the predominant protein [7]. In contrast, in the milk of infected goats, cathelicidin-2 was found to be the predominant protein of the family.

In previous works, the presence of cathelicidin proteins in mammary epithelial cells in goats was not confirmed [8,25]. The present study provides indirect evidence for this; we postulate that the early (4 h post-challenge, i.e., before the influx of neutrophils into the mammary tissue) detection of the proteins occurred consequently to the destruction of the mammary epithelial cells by the invading staphylococci, whence the cathelicidin proteins found in milk originated. This hypothesis is directly similar to relevant findings previously reported in sheep [5]. The subsequent increase in the spot densities of the proteins at later sampling points might signal their origination from the neutrophil influx that entered the mammary gland following the bacterial infection, as previously reported [8,25]. The isolation of antimicrobial peptides from goat leucocytes in previous relevant studies [26,27,28] lends support to this suggestion.

The increased overall accuracy of the method can result in a reliable diagnosis of mastitis in individual goats. Subsequently, this will support the early instigation of correct treatment of the affected animals, thus improving animal health and welfare and also reducing the risk of dissemination of the pathogen within the farm [6,29,30]. The increased correlation of the detection of cathelicidin proteins with presence of mastitis at the early stages of the infection supports the use of these proteins as a biomarker for mastitis in goats.

The use of a small number of goats in the experimental study can be considered as a potential limitation of the present study. In this respect, it is noted that the official animal welfare monitoring board imposed an upper limit in the number of animals that were to be used in the study, in order to safeguard the welfare of the experimental animals, given than mastitis can be a painful infection in small ruminants. There was however a clear consistency in results, which was observed across all the experimental goats and in all sampling points of the experiment. It has been documented that the number of animals used in a project must be as small as possible to obtain the required results; in this respect, the use of repeated measurements in the same animals can contribute to minimizing the number of animals included in an experiment, especially if the analysis shows statistical significance. This way, the use of larger number of animals can be avoided, maintaining welfare standards [31]. This can be coupled with similar results previously reported in sheep. Moreover, a post hoc analysis indicated that the power of this study in comparison to previous results carried out under identical conditions was 75.1%. Although the use of retrospective analysis techniques is controversial, the result may be considered to confirm that the findings supported the usefulness of the diagnostic procedure. All the above should be aligned with the need to safeguard the welfare of experimental animals [32], which must be a priority for researchers carrying out work with experimental animals; ultimately, all these justified the restrictions imposed by the competent authorities without greatly reducing the quality of the results. Moreover, it is noted that small animal numbers have been commonly used in proteomics work on small ruminant mastitis, comparably to those used in the current study. Such studies employ serial sample collection (i.e., repeated samplings after intervention), just as in the current study. That way, each animal can act as its own control and deliver a within-subject statistical dimension that markedly increases power relative to a cross-sectional design. Further, ethical regulations in the European Union and the national authorities in Greece explicitly favour a reduction in animal use when omics platforms can extract large datasets from limited material. For these reasons, a three-goat design was authorized for the current experimentation because it fully aligned with accepted practice in proteomics studies and with high animal welfare standards, while still generating biologically meaningful and valuable proteomics data.

The use of only one mastitis-causing organism (specifically, *S. simulans*) may also be viewed as another possible limitation. Nevertheless, cathelicidin proteins are non-specific antibacterial proteins [33,34,35,36,37]. In the past, Cubeddu et al. [5] reported the detection of cathelicidin in cases of ovine mastitis caused by *Staphylococcus aureus*, *Streptococcus uberis,* or *Mycoplasma agalactiae*; more recently, Katsafadou et al. [7] detected cathelicidin in the milk of ewes after intramammary challenge with *S. chromogenes* or *Mannheimia haemolytica*. Moreover, Addis et al. [38] reported that the identity of the mastitis causal agent ‘did not compromise diagnostic performance’ of the detection of cathelicidin in milk.

### 4.2. Farm Study

The results in the bulk-tank milk samples collected from the farms did not provide any support for using the methodology as a means for the diagnosis of mastitis at the farm level. Although the prevalence of mastitis in dairy goat and sheep farms in Greece varies between 20% and 30% [39,40], no cathelicidin proteins were detected in the bulk-tank milk.

One reason for this may be simply a dilution effect; milk from the few recent cases of mastitis on a farm would be diluted within the larger amount of milk produced by the healthy animals on the farm, which would make the detection of the cathelicidin proteins impossible. In this context, one cannot rule out that the concentration of cathelicidin proteins in the bulk-tank milk was below the detection threshold of the 2-DE technique. Coomassie blue typically detects proteins at concentrations of ≥30 to 100 ng per spot [41]. Alternatively, one may suggest that farmers might be milking animals with mastitis separately; that way, their milk would not be mixed with that of healthy animals, avoiding increases in the bulk-tank milk bacterial counts, somatic cell counts, and antibiotic residues. Another reason might be the absence of sufficient numbers of animals with early-stage mastitis when presence of cathelicidin proteins in the milk is high; as observed in the experimental study, the concentration of these proteins decreases with the advancement of the infection; therefore, it becomes difficult to detect it and even more so within the pooled bulk-tank milk of the whole herd.

It is interesting that in the international literature, all previous studies on the use of cathelicidin proteins for the diagnosis of mastitis, performed by other workers in cattle [38,42], in sheep [5] and in buffaloes [43], were performed in individual animals. The lack of published results obtained at the farm level may possibly reflect the production of negative results by previous researchers, who, nevertheless, may have opted not to share them.

### 4.3. Prospects

The consistency of the results among the experimental animals, coupled with the previous findings of the detection of cathelicidin proteins in the milk of ewes with mastitis, offers a substantial advantage for use of the findings in sheep and goat farms alike. A potential exploitation of the findings could be the development of a detector of cathelicidin proteins at the individual animal level, for future incorporation into milking systems installed on farms. The difference between goats and sheep in the predominant cathelicidin protein present in the milk of infected animals, and given that milking systems for sheep and goat farms have a similar design, suggests that such a detector would need to be regulated for both cathelicidin-1 and cathelicidin-2 to work independently of the animal species milked (and also to account for the fact that there also mixed sheep–goat farms, where all animals are milked at the same parlour). In any case, the detection of these proteins as early as four hours after challenge indicates that, in a 12 h milking cycle, infections that had occurred at the latest 16 h prior to sampling can be detected through this approach.

The findings contribute to the improvement of the diagnosis of mastitis in goats. There is interest in early and accurate diagnosis of the infection in goats, given its consequences for animal production [24,44], as well as the potential for transferring zoonotic pathogens to the food chain [45,46].

This methodology offers a significant advantage over the evaluation of somatic cell counts, which have been shown to increase later than cathelicidin proteins and to reach figures indicative of mastitis much later. Moreover, the detection of cathelicidin proteins is qualitative (with their presence in milk indicative of mastitis), with no need to quantify their amount or set a threshold for detection in the milk samples.

## 5. Conclusions

The detection of cathelicidin biomolecules can offer increased accuracy for the early detection of mastitis in goats. Post-infection, the detection of cathelicidin proteins in the milk of individual animals occurred earlier than the increase in somatic cell counts in milk from the same animals. This approach presents an advantage in that cathelicidin proteins can be used as a non-specific biomarker, as simply a ‘positive’/‘negative’ assessment would be sufficient.

## Figures and Tables

**Table 1:** Median (interquartile range) spot optical densities of cathelicidin biomolecules on 2-DE gels obtained from sequential milk whey samples from mammary glands inoculated with *S. simulans* for 48 h subsequently to challenge.

Cathelicidin Proteins ^1^	Mammary Gland ^2^	Timepoint of the Study ^3^										
		D(−1)	D(0)	D(0+ 4 h)	D(0+ 8 h)	D(0+ 12 h)	D(0+ 16 h)	D(0+ 20 h)	D(1)	D(1+ 8 h)	D(1+ 16 h)	D(2)
CHTL-1	chall.	0.0 (0.0)	0.0 (0.0)	691.1 (379.0)	1847.7 (1108.1)	2594.5 (904.2)	2421.5 (394.5)	1344.4 (153.9)	919.8 (36.4)	613.3 (97.4)	523.3 (154.3)	452.4 (66.0)
	contr.	0.0 (0.0)	0.0 (0.0)	0.0 (0.0)	0.0 (0.0)	0.0 (0.0)	0.0 (0.0)	0.0 (10.7)	15.0 (13.5)	24.5 (15.1)	24.1 (13.8)	24.3 (13.3)
CHTL-2	chall.	0.0 (0.0)	0.0 (0.0)	304.8 (1237.1)	4310.8 (3679.6)	2123.8 (6352.5)	3631.7 (2627.1)	2249.7 (1245.0)	1030.3 (1609.8)	761.9 (1236.4)	502.1 (612.5)	436.3 (437.1)
	contr.	0.0 (0.0)	0.0 (0.0)	0.0 (0.0)	0.0 (0.0)	0.0 (0.0)	0.0 (0.0)	0.0 (0.0)	0.0 (4.7)	0.0 (6.8)	0.0 (12.8)	0.0 (11.1)

^1^: CHTL: cathelicidin; ^2^: chall.: inoculated gland, contr.: control gland; ^3^: D(0): timepoint for intramammary inoculation of does with *S. simulans*.

**Table 2:** A 2 × 2 contingency table indicating number of milk samples from mammary glands with or without mastitis [‘positive’ (+) or ‘negative’ (−)] in relation to detection of cathelicidin biomolecules in these samples [‘positive’ (+) or ‘negative’ (−)].

		Presence of Mastitis	
		+	–
**Detection of Cathelicidin Proteins**	+	25	12
	–	0	29

**Table 3:** Mean somatic cell counts and chemical composition (fat, total proteins) in the bulk-tank milk from 32 goat farms and 57 sheep farms.

	Goat Farms	Sheep Farms
Somatic cell counts (cells mL^−1^)	0.614 × 10^6^ (0.467 × 10^6^ –0.808 × 10^6^)	0.517 × 10^6^ (0.415 × 10^6^ –0.643 × 10^6^)
Fat (%)	4.70 ± 0.15	6.15 ± 0.10
Total proteins (%)	3.17 ± 0.02	4.50 ± 0.02

## Data Availability

Most data associated with this study are provided in the manuscript. The remaining data are available on request from the corresponding author. The data are not publicly available as they form part of the Ph.D. thesis of the first author, which has not yet been examined, approved and uploaded in the official depository of Ph.D. theses from Greek Universities.

## Supplementary Materials

The following supporting information can be downloaded at https://www.mdpi.com/article/10.3390/ani15152301/s1, Figure S1: 2-DE gels with annotation of cathelicidin-1 and cathelicidin-2, obtained from milk samples (whey) collected from the mammary glands of a doe before or after intramammary challenge with *Staphylococcus simulans*; Figure S2: Trendlines for spot optical densities of cathelicidin-1 (blue dashed line) or cathelicidin-2 (red dashed line) on 2-DE gels obtained from sequential milk whey samples from mammary glands inoculated with *S. simulans* and comparison with trendline of California Mastitis Test scores (black dotted line) in the same samples.

## References

[1] McDonald T.L., Larson M.A., Mack D.R., Weber A. (2001). Elevated extrahepatic expression and secretion of mammary-associated serum amyloid A 3 (M-SAA3) into colostrum. Vet. Immunol. Immunopathol..

[2] Addis M.F., Pisanu S., Marogna G., Cubeddu T., Pagnozzi D., Cacciotto C., Campesi F., Schianchi G., Rocca S., Uzzau S. (2013). Production and release of antimicrobial and immune defense proteins by mammary epithelial cells following *Streptococcus uberis* infection of sheep. Infect. Immun..

[3] Addis M.F., Tedde V., Dore S., Pisanu S., Puggioni G.M.G., Roggio A.M., Pagnozzi D., Lollai S., Cannas E.A., Uzzau S. (2016). Evaluation of milk cathelicidin for detection of dairy sheep mastitis. J. Dairy Sci..

[4] Katsafadou A.I., Tsangaris G.T., Anagnostopoulos A.K., Billinis C., Barbagianni M.S., Vasileiou N.G.C., Spanos S.A., Mavrogianni V.S., Fthenakis G.C. (2019). Differential quantitative proteomics study of experimental *Mannheimia haemolytica* mastitis in sheep. J. Proteom..

[5] Cubeddu T., Cacciotto C., Pisanu S., Tedde V., Alberti A., Pittau M., Dore S., Cannas A., Uzzau S., Rocca S. (2017). Cathelicidin production and release by mammary epithelial cells during infectious mastitis. Vet. Immunol. Immunopathol..

[6] Mavrogianni V.S., Menzies P.I., Fragkou I.A., Fthenakis G.C. (2011). Principles of mastitis treatment in sheep and goats. Vet. Clin. N. Am. Food Anim. Pract..

[7] Katsafadou A.I., Tsangaris G.T., Vasileiou N.G.C., Ioannidi K.S., Anagnostopoulos A.K., Billinis C., Fragkou I.A., Papadopoulos E., Mavrogianni V.S., Michael C.K. (2019). Detection of Cathelicidin-1 in the Milk as an Early Indicator of Mastitis in Ewes. Pathogens.

[8] Olumee-Shabon Z., Swain T., Smith E.A., Tall E., Boehmer J.L. (2013). Proteomic analysis of differentially expressed proteins in caprine milk during experimentally induced endotoxin mastitis. J. Dairy Sci..

[9] Reczyńska D., Witek B., Jarczak J., Czopowicz M., Mickiewicz M., Kaba J., Zwierzchowski L., Bagnicka E. (2019). The impact of organic vs. inorganic selenium on dairy goat productivity and expression of selected genes in milk somatic cells. J. Dairy Res..

[10] Tedde V., Bronzo V., Puggioni G.M.G., Pollera C., Casula A., Curone G., Moroni P., Uzzau S., Addis M.F. (2019). Milk cathelicidin and somatic cell counts in dairy goats along the course of lactation. J. Dairy Res..

[11] Tsugami Y., Nii T., Isobe N. (2023). Valine treatment enhances antimicrobial component production in mammary epithelial cells and the milk of lactating goats without influencing the tight junction barrier. J. Mammary Gland Biol. Neoplasia.

[12] Sun J.K., Liang Z.L., Nii T., Suzuki N., Isobe N. (2025). Antimicrobial component concentrations in the milk of peripartum goats. Anim. Sci. J..

[13] Fthenakis G.C., Jones J.E.T. (1990). The effect of inoculation of coagulase-negative staphylococci into the ovine mammary gland. J. Comp. Pathol..

[14] Miles A.A., Misra J.S. (1938). The estimation of the bactericidal power of the blood. J. Hyg. Camb..

[15] Vasileiou N.G.C., Chatzopoulos D.C., Cripps P.J., Ioannidi K.S., Gougoulis D.A., Chouzouris T.M., Lianou D.T., Calvo Gonzalez-Valerio T., Guix Vallverdu R., Argyros S. (2019). Evaluation of efficacy of a biofilm-embedded bacteria-based vaccine against staphylococcal mastitis in sheep—A randomized, placebo-controlled field study. J. Dairy Sci..

[16] Barrow G.I., Feltham R.K.A. (1993). Manual for the Identification of Medical Bacteria.

[17] Euzeby J.P. (1997). List of bacterial names with standing in nomenclature: A folder available on the Internet. Int. J. Syst. Bacteriol..

[18] Schalm O.W., Carroll E.J., Jain N.C. (1971). Bovine Mastitis.

[19] European Food Safety Authority (2014). Scientific opinion on the welfare risks related to the farming of sheep for wool, meat and milk production. EFSA J..

[20] Lianou D.T., Michael C.K., Vasileiou N.G.C., Petinaki E., Cripps P.J., Tsilipounidaki K., Katsafadou A.I., Politis A.P., Kordalis N.G., Ioannidi K.S. (2021). Extensive countrywide field investigation of somatic cell counts and total bacterial counts in bulk-tank raw milk in goat herds in Greece. J. Dairy Res..

[21] Anagnostopoulos A.K., Katsafadou A.I., Pierros V., Kontopodis E., Fthenakis G.C., Arsenos G., Karkabounas S.C., Tzora A., Skoufos I., Tsangaris G.T. (2016). Milk of Greek sheep and goat breeds; characterization by means of proteomics. J. Proteom..

[22] Wiggans G.R., Shook G.E. (1987). A lactation measure of somatic cell count. J. Dairy Sci..

[23] Franzoi M., Manuelian C.L., Penasa M., De Marchi M. (2020). Effects of somatic cell score on milk yield and mid-infrared predicted composition and technological traits of Brown Swiss, Holstein Friesian, and Simmental cattle breeds. J. Dairy Sci..

[24] Tibebu A., Teshome Y., Tamrat H., Bahiru A. (2025). Mastitis in goat: A review of etiology, epidemiology, economic impact, and public health concerns. One Health.

[25] Zhang G.W., Lai S.J., Yoshimura Y., Isobe N. (2014). Expression of cathelicidins mRNA in the goat mammary gland and effect of the intramammary infusion of lipopolysaccharide on milk cathelicidin-2 concentration. Vet. Microbiol..

[26] Shamova O., Orlov D., Stegemann C., Czihal P., Hoffmann R., Brogden K., Kolodkin N., Sakuta G., Tossi A., Sahl H.G. (2009). ChBac3.4: A novel proline-rich antimicrobial peptide from goat leukocytes. Int. J. Pept. Res. Ther..

[27] Sharma A., Kumar A., Nigam R., Pandey V., Singh P. (2020). A minireview on antimicrobial peptides of goats and their role in host defense. Biosc. Biotech. Res. Comm..

[28] Yan Y., Zhu K., Liu H., Fan M., Zhao X., Pan M., Ma B., Wei Q. (2023). The relationship between mastitis and antimicrobial peptide S100A7 expression in dairy goats. Vet. Sci..

[29] Milner P., Page K.L., Hillerton J.E. (1997). The effects of early antibiotic treatment following diagnosis of mastitis detected by a change in the electrical conductivity of milk. J. Dairy Sci..

[30] Laven R. (2010). Mastitis Part 4—Detecting and Treating Clinical Mastitis. NADIS Animal Health Skills. https://www.nadis.org.uk/disease-a-z/cattle/mastitis/mastitis-part-4-detecting-and-treating-clinical-mastitis/.

[31] National Health and Medical Research Council (2013). Australian Code for the Care and Use of Animals for Scientific Purposes.

[32] Festing M.F.W. (2011). How to Reduce the Number of Animals Used in Research by Improving Experimental Design and Statistics. ANZCCART Fact Sheet T10. https://anzccart.adelaide.edu.au/ua/media/553/reduce-numbers-in-animals.pdf.

[33] Kosciuczuk E.M., Lisowski P., Jarczak J., Strzałkowska N., Jozwik A., Horbanczuk J., Krzyzewski J., Zwierzchowski L., Bagnicka E. (2012). Cathelicidins: Family of antimicrobial peptides. A review. Mol. Biol. Rep..

[34] Roby K.D., Nardo A.D. (2013). Innate immunity and the role of the antimicrobial peptide cathelicidin in inflammatory skin disease. Drug Discov. Today Dis. Mech..

[35] van Harten R.M., van Woudenbergh E., van Dijk A., Haagsman H.P. (2018). Cathelicidins: Immunomodulatory antimicrobials. Vaccines.

[36] Xie F., Zan Y., Zhang X., Zhang H., Jin M., Zhang W., Zhang Y., Liu S. (2020). Differential abilities of mammalian cathelicidins to inhibit bacterial biofilm formation and promote multifaceted immune functions of neutrophils. Int. J. Mol. Sci..

[37] Guerra M.E.S., Vieira B., Calazans A.P.C.T., Destro G.V., Melo K., Rodrigues E., Waz N.T., Girardello R., Darrieux M., Converso T.R. (2024). Recent advances in the therapeutic potential of cathelicidins. Front. Microbiol..

[38] Addis M.F., Bronzo V., Puggioni G.M.G., Cacciotto C., Tedde V., Pagnozzi D., Locatelli C., Casula A., Curone G., Uzzau S. (2017). Relationship between milk cathelicidin abundance and microbiologic culture in clinical mastitis. J. Dairy Sci..

[39] Kalogridou-Vassiliadou D. (1991). Mastitis-related pathogens in goat milk. Small Rumin. Res..

[40] Vasileiou N.G.C., Cripps P.J., Ioannidi K.S., Chatzopoulos D.C., Gougoulis D.A., Sarrou S., Orfanou D.C., Politis A., Calvo Gonzalez-Valerio T., Argyros S. (2018). Extensive countrywide field investigation of subclinical mastitis in sheep in Greece. J. Dairy Sci..

[41] Simpson R.J. (2010). Rapid coomassie blue staining of protein gels. Cold Spring Harb. Protoc..

[42] Addis M.F., Tedde V., Puggioni G.M.G., Pisanu S., Casula A., Locatelli C., Rota N., Bronzo V., Moroni P., Uzzau S. (2016). Evaluation of milk cathelicidin for detection of bovine mastitis. J. Dairy Sci..

[43] Puggioni G.M.G., Tedde V., Uzzau S., Guccione J., Ciaramella P., Pollera C., Moroni P., Bronzo V., Addis M.F. (2020). Evaluation of a bovine cathelicidin ELISA for detecting mastitis in the dairy buffalo: Comparison with milk somatic cell count and bacteriological culture. Res. Vet. Sci..

[44] Modi R.J., Patel N.M., Patel Y.G., Islam M.M., Nayak J.B., Rana T. (2024). Goat farming: A boon for economic upliftment. Trends in Clinical Diseases, Production and Management of Goats.

[45] Gallo M., Ferrara L., Calogero A., Montesano D., Naviglio D. (2020). Relationships between food and diseases: What to know to ensure food safety. Food Res. Int..

[46] Aliyi M.B. (2023). Review on goat mastitis and associated bacterial zoonoses in raw milk from mastitis infected dairy goat. Adv. Dairy Sci. Res..

