# Supplementary material for: Detection of Cathelicidin-1 and Cathelicidin-2 Biomolecules in the Milk of Goats and Their Use as Biomarkers for the Diagnosis of Mastitis
Source: Animals (Basel). 2025 Aug 6;15(15):2301. doi: 10.3390/ani15152301 (PMC12345514; doi:10.3390/ani15152301)
Supplement: Supplementary file 1 [file animals-15-02301-s001.zip › animals-3704901-supplementary material.pdf]

Detection of Cathelicidin-1 and Cathelicidin-2 Biomolecules in the Milk of Goats and Their Use as Biomarkers for the Diagnosis of Mastitis

M.V. Bourganou, D.V. Liagka, K. Vougas, D.T. Lianou, N.G.C. Vasileiou, K.S. Dimoveli, A.P. Politis, N.G. Kordalis, E. Petinaki, V.S. Mavrogianni, G.Th. Tsangaris, G.C. Fthenakis, and A.I. Katsafadou

Figure S1. 2-DE gels with annotation of cathelicidin-1 and cathelicidin-2, obtained from milk samples (whey) collected from the mammary glands of a doe before or after intramammary challenge with *Staphylococcus simulans* (protein identification by MALDI-TOF MS).

(a) 2-DE gel obtained from a whey sample, from the mammary gland to be inoculated, but before challenge; the area in red indicates the region of the gels shown in detail in (b) and (c).

(b) Region of 2-DE gels obtained from whey samples, from the inoculated mammary gland, before or sequentially after challenge; from top left to the right and from bottom right to the right: before inoculation (D0), 4 h after inoculation (D0+4 h), 8 h after inoculation (D0+8 h), 12 h after inoculation (D0+12 h), 16 h after inoculation (D0+16 h) and 24 h after inoculation (D1).

(a)

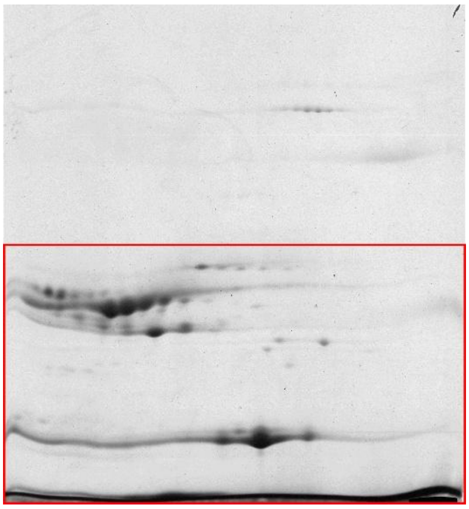

(b)

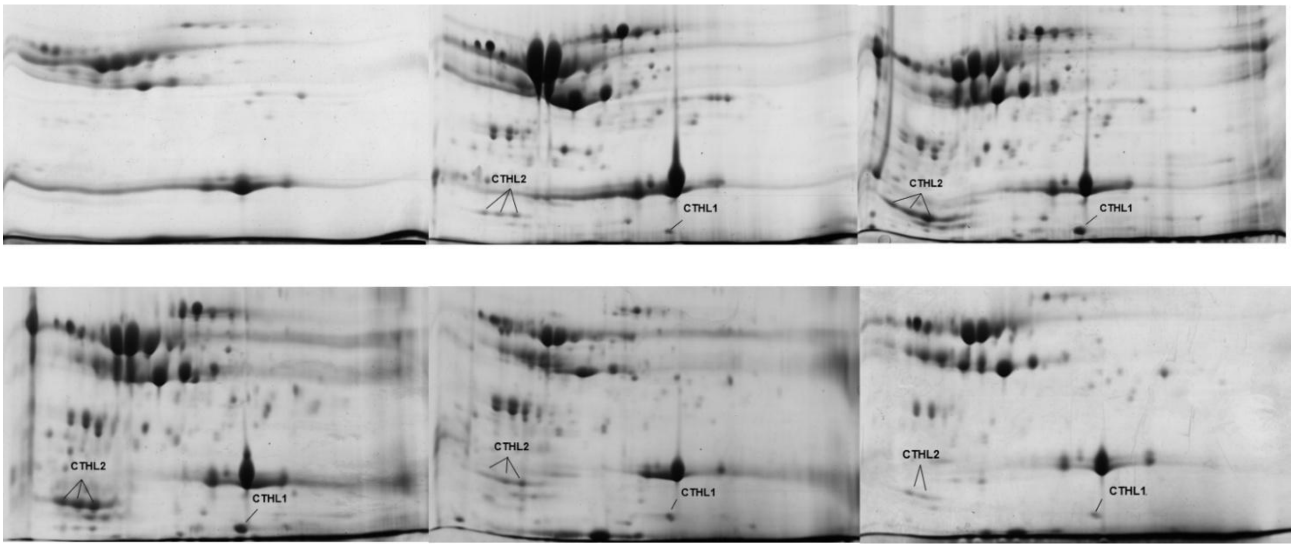

Horizontal axis: isoelectric point 4 to 7 from left to right; vertical axis: molecular weight 10 to 100 kDa from bottom to top.

Figure S2. Trendlines for spot optical densities of cathelicidin-1 (blue dashed line) or cathelicidin-2 (red dashed line) on 2-DE gels obtained from sequential milk whey samples from mammary glands inoculated with *S. simulans* and comparison with trendline of California Mastitis Test scores (black dotted line) in the same samples; trendlines for the spot optical densities of the two cathelicidin proteins indicated an early increase in the values, which reached maximum figures after 12 to 24 h post-challenge, in contrast with California Mastitis Test scores, which increased at a later stage and continued to rise as the infection developed further.

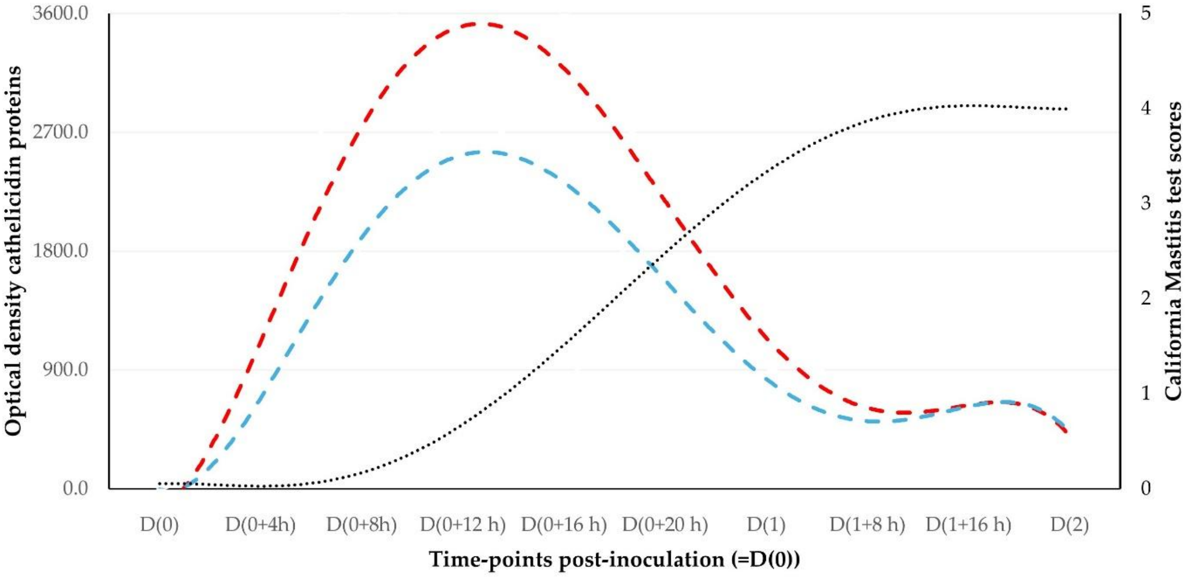

Value 0 corresponds to score 'negative', value 1 corresponds to score 'trace', value 2 corresponds to score '1', value 3 corresponds to score '2', and value 4 corresponds to score '3' (value 5 shown only for the purposes of constructing the graphs).
